# Whole genome analysis of selection associated with resistance to heat stress in chickens

**DOI:** 10.1038/s41598-026-41813-8

**Published:** 2026-04-07

**Authors:** Sevda Hosseinzadeh, Seyed Abbas Rafat, Arash Javanmard, Karim Hasanpur, Philippe Bardou, Mathieu Charles, Christophe Klopp, Adrian L. Smith, Steven R. Fiddaman

**Affiliations:** 1 Department of Animal Science, Faculty of Agriculture, University of Tabriz, Tabriz, Iran; 2 Sigenae, GenPhySE, Université de Toulouse, INRAE, ENVT, Castanet Tolosan, F-31326 France; 3 GABI, SIGENAE, AgroParisTech, INRAE, Université Paris-Saclay, Jouy-en‐Josas, France; 4 INRAE, BioinfOmics, Université Fédérale de Toulouse, GenoToul Bioinformatics facility, Sigenae, Castanet-Tolosan, 31326 France; 5 Department of Biology, Sir Peter Medawar Building, University of Oxford, Oxford, OX1 3SZ UK; 6 The Pirbright Institute, Ash Rd, Pirbright, Woking, GU24 0NF UK

**Keywords:** Chicken, Heat tolerance, Selection, Whole genome, Adaptation, Evolution, Genetics

## Abstract

Following their domestication, chickens were translocated around the world to novel environments. Through a combination of natural and artificial selection, chickens adapted to these local conditions, creating significant genetic diversity across populations worldwide. Studying this diversity in the context of local environmental conditions may offer insights into mechanisms of adaptation to environmental stressors. In this study, we analyzed genomic data from the Chicken Genomic Diversity Consortium, applying multiple statistical approaches, including fixation index (F_ST_), nucleotide diversity (π), Tajima’s D, and runs of homozygosity (ROH), to identify selective sweeps among indigenous chickens from Afghanistan, China, Indonesia, Iran and Pakistan, compared with White Leghorn chickens. We identified sweeps in 14 genes related to heat tolerance, associated with relevant gene ontology (GO) terms and located within ROH regions. These genes, such as *CDH23*, *NPSR1*, *MCU*, *TRPV2*, *TRPV1*, *TRPV3*, *ATP2B4*, *CALM1*, *CACNB2*, *TRAT1*, *BDNF*, *SCIN*, *WIPF3*, *PRKD1*, and *DNAJC10* play crucial roles in calcium signaling pathways, thermal sensation, and the plasticity of neurodevelopmental processes. These findings illustrate the significant role of selection in shaping genomic differentiation across chicken populations and provide insights into the genetic basis of adaptation to environmental stressors.

**Supplementary Information:**

The online version contains supplementary material available at 10.1038/s41598-026-41813-8.

## Introduction

Heat stress (HS) is an important challenge in the poultry industry^1^, especially in warm climates, causing substantial economic losses in both layer and broiler farms. In recent decades, global climate data has shown a clear warming trend across much of the world^2^, amplifying the impact of climate on poultry production. The Temperature Humidity Index (THI) is widely used to evaluate how environmental conditions impact poultry, serving as a measure of livestock productivity in relation to climate factors^3^. This index reflects the external forces that shift an animal’s body temperature from its normal range, providing a useful indicator of climate stress on livestock^4, 5^. Chickens’ lack of sweat glands^6^, insulating feather cover, and the high density in commercial rearing further exacerbate their susceptibility to HS^7^. Exposure to high temperatures disrupts several physiological processes, including immune function^8^, metabolism^9^, oxidative balance^10^, hemodynamics^11^, and protein synthesis^12^, often leading to reduced feed intake, lower productivity, and higher morbidity and mortality^13^.

Over time, indigenous chickens adapt to local environmental conditions through natural selection, detected genetically via distinctive genomic signatures in selected regions. These include increased frequency of selected alleles^14^, extended linkage disequilibrium^15^, increased homozygosity^16^, and reduced local genetic diversity^17^. To identify selection signatures, various statistical methods are used, grouped into two main categories: those analyzing variation within a population (intra-population) and those examining variation between populations (inter-population). Within-population approaches include metrics like runs of homozygosity (ROH) to detect long segments of homozygosity^16^, nucleotide diversity (π) to assess genetic variation levels^18^, and Tajima’s D to evaluate deviations from neutral evolution^19^. In contrast, between-population studies often rely on metrics like the fixation index (F_ST_)^20^, which measures genetic differentiation between populations and helps highlight selection patterns that may differ across groups.

To investigate mechanisms of adaptation to heat stress, we carried out genomic analyses on indigenous chickens from Afghanistan, China (covering Huaixiang, Huiyang, Huaibeima, Jianghan, and Wuhua regions), Indonesia (Bali), Iran (Shiraz and Zahedan), and Pakistan, comparing these with White Leghorn chickens. Commercial chickens – like the White Leghorn – have been subject to intensive artificial selection, and are highly susceptible to HS due to elevated metabolic demands and extensive selection for high egg production, which often comes at the expense of traits like heat tolerance^7^. Comparing indigenous breeds to the White Leghorn (a lineage selected to exist in temperate environments with a known susceptibility to HS) provides crucial insights into the mechanisms of adaptation to HS, paving the way for breeding strategies aimed at improving heat tolerance while preserving productivity^21^.

## Materials and methods

### Whole Genome Dataset

The dataset included 49 commercial White Leghorn chickens and indigenous chickens selected from locations with a (THI) above 72, which is commonly regarded as the threshold at which heat stress begins to affect poultry^22, 23^. The indigenous chicken dataset (*n* = 152) comprised chickens from 10 different locations experiencing high THI, with at least 10 chickens from each group (Pakistan [*n* = 30], Shiraz [*n* = 16)], Zahedan [*n* = 19], Afghanistan [*n* = 11], Bali [*n* = 15], Huaixiang [*n* = 10], Huiyang [*n* = 10], Huaibeima [*n* = 10], Jianghan (*n* = 10), and Wuhua (*n* = 21)). Additional details on these samples are provided in **Supplementary Table ****S1**. After excluding variants with low quality, high missing rates, and low frequencies, a total of 15,325,788 SNPs were retained for analysis across the populations.

The genomes used in this study were curated by the Chicken Genomic Diversity Consortium^24^ using a standardized pipeline detailed in **Supplementary information**,** Fig. ****S1**. All samples were mapped to the bGalGal1.mat.broiler.GRCg7b reference genome. To minimize potential bias due to missing or unreliable genotypes, we filtered the VCF files for each commercial and indigenous chicken group. Filtering criteria included the removal of indels, SNP call rate, minor allele count (MAC), genotype quality, and minimum and maximum depth. This was performed using VCFtools with the following options: --remove-indels --max-alleles 2 --minGQ 20 --minDP 8 --maxDP 150 --max-missing 0.9 --mac 5.

### Temperature-Humidity Index (THI)

To categorize indigenous chickens based on their local environmental conditions, we calculated a Temperature-Humidity Index (THI) for each population’s geographic coordinates, identifying those populations residing in regions characterized by high heat stress based on the hottest season of the year.

The average temperature and humidity percentages for each location were based on historical weather reports collected from (https://www.timeanddate.com) over the period from 1992 to 2021, using coordinate data to calculate the THI. Additionally, the average temperature and humidity percentages associated with the hottest month of the year were used to calculate a THI.

The equation used to calculate the THI was as follow^25^:

$$\:THI=\left(1.8\:\times\:\:{T}_{avg}+32\right)-(0.55-0.0055\times\:{RH}_{avg})\times\:(1.8\times\:{T}_{avg}-26)$$

$\:{T}_{avg}$ = The average air temperature (˚C).

$\:{RH}_{avg}$= The average relative humidity (%).

### Population structure

Principal Component Analysis (PCA) was conducted to examine genetic variation between populations of indigenous chickens and the Leghorn population, utilizing PLINK V1.9^26^ software. Genotypes for PCA clustering were pruned based on linkage disequilibrium (LD) using PLINK with the option --indep-pairwise 50 10 0.1.

### Exploration of selective sweep regions

We used three statistical methods—genetic differentiation index (F_ST_), nucleotide diversity (π), and Tajima’s D—to identify selective sweeps in pairs of indigenous chickens and White leghorn chickens. Our sample pairs include: White Leghorn with indigenous chickens collected from Iran (Shiraz and Zahedan), Afghanistan, Indonesia (Bali), China (Huaixiang, Huiyang, Huaibeima, Jianghan, and Wuhua), and Pakistan.

We used the PopGenome R package (version 2.7.5)^27^ to calculate F_ST_ nucleotide diversity, and Tajima’s D with 100 Kb sliding windows and 10 Kb step size for each population pair separately.

To identify candidate regions under selective sweeps, we applied a multi-step filtering strategy. Initially, we selected genomic windows containing at least five single nucleotide polymorphisms (SNPs), suggesting potential regions of interest. We then evaluated genetic differentiation (F_ST_) between White Leghorn and indigenous chicken breeds, retaining windows that ranked in the top 5% of F_ST_ values. From these, we focused on windows shared in at least 50% of the pairwise comparisons between Leghorn and local breeds. Next, we analyzed nucleotide diversity and Tajima’s D by comparing the values of θπ and Tajima’s D between indigenous and White Leghorn chickens. We selected windows falling within the top and bottom 2.5% of the distributions of log₂ (θπ_indigenous/θπ_WhiteLeghorn) and (Tajima’s D_indigenous – Tajima’s D_WhiteLeghorn), respectively. Finally, we refined the analysis by including only those windows shared across at least 50% of the indigenous populations for both nucleotide diversity and Tajima’s D metrics.

### Annotation of genetic variants

Genomic regions selected as candidate sweeps based on F_ST_, log₂ (θπ_indigenous/θπ_WhiteLeghorn) and (Tajima’s D_indigenous – Tajima’s D_WhiteLeghorn) values were annotated using the GRCg7a genome reference (https://ftp.ensembl.org/pub/release113/gtf/gallus_gallus/Gallus_gallus.bGalGal1.mat.broiler.GRCg7b.113.gtf.gz). This annotation process, facilitated by the “rtracklayer"^28^ and “GenomicRanges"^29^ R packages (R version 4.2.3), and SnpEff^30^ software enabled the identification of transcripts.

### Characteristics of Runs of homozygosity (ROH)

This study examined runs of homozygosity (ROH), an indicator of genomic autozygosity, in 10 indigenous chicken populations and White Leghorn populations by analyzing extended segments of homozygous SNPs inherited from a recent common ancestor. ROH were calculated using PLINK v1.9 with the parameters: --homozyg, --homozyg-group, --homozyg-kb 300, --homozyg-window-snp 50, --homozyg-density 100, --homozyg-gap 1000, --homozyg-window-het 3, --homozyg-window-threshold 0.05, and --homozyg-window-missing 3.

The inbreeding coefficient (F) was calculated for each individual and across each chicken group using VCFtools and PLINK’s –het option. Detected ROHs were classified into three length categories: 0 to < 1 Mb, 1 to < 3 Mb, and > 3 Mb.

### Gene set enrichment and pathway analysis

To identify candidate genes and nearly fixed alleles associated with heat tolerance, we focused on genes that were significant in at least three of the following analyses: F_ST_, θπ, Tajima’s D, and ROH. Heat tolerance-related candidate genes were further identified based on our previous findings^31, 32^, and genes listed in the NCBI database (https://www.ncbi.nlm.nih.gov/ge). We then constructed a PPI network for these common genes using the STRING database (https://string-db.org/) to identify hub genes within the network. For gene ontology (GO) pathway enrichment analysis, we used the ClueGO plugin in Cytoscape, considering GO terms and pathways as significantly enriched if they had Bonferroni-corrected p-values < 0.05.

### Genotype extraction and allele frequency analysis

SNP data for 14 target genes were extracted from a VCF file using R (version 4.2.2), with packages vcfR^33^, dplyr^34^, tidyr^35^, and readr^36^. Allele frequencies were calculated separately for the indigenous and White Leghorn populations. For each SNP, the counts of reference and alternative alleles were obtained, and fixation status was determined. Fisher’s exact test was applied to assess significant differences in allele frequencies between populations, and p-values were adjusted using false discovery rate (FDR) correction.

## Results

### Population structure

**Fig. 1:**
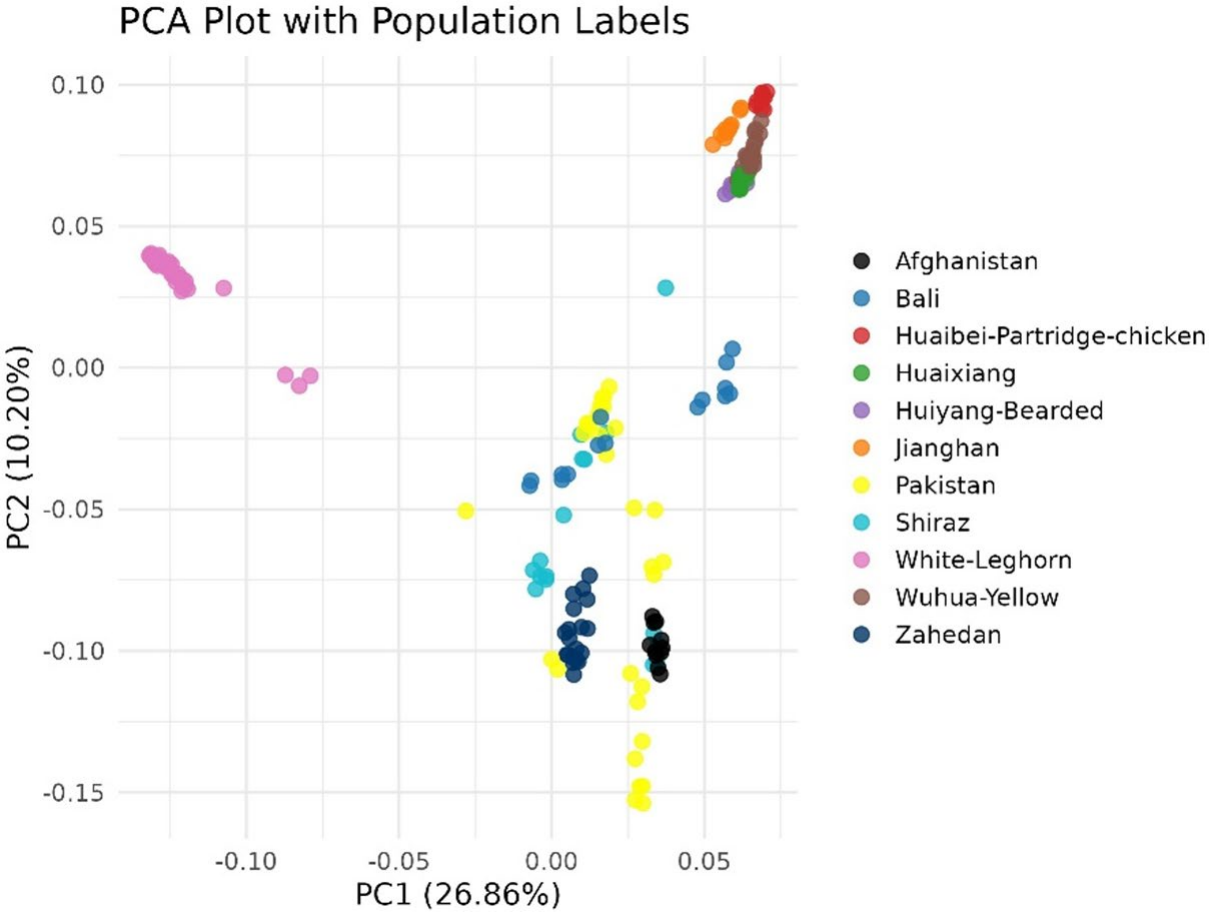
Principal component analysis (PCA) reveals an east-west cline in indigenous chicken relatedness, and differentiation between indigenous chickens and commercial White Leghorns.

Principal component analysis revealed significant population structure among the chicken populationsn (Fig. 1). The commercial White Leghorn chickens formed a distinct cluster that was separated from all indigenous breeds along PC1, suggesting substantial differentiation between commercial and indigenous chickens. Furthermore, indigenous chickens demonstrated an east-west cline in relatedness along PC2, highlighting genetic differentiation between eastern (Chinese) indigenous chickens vs. chickens from Afghanistan, Iran and Pakistan.

### Genome‑wide selective sweep analysis

To identify potential selective sweeps, we analyzed the genome for regions with high differentiation levels between White Leghorn compared to each indigenous chicken population separately. We identified 3,042 genomic windows with F_ST_ values in the top 5% in at least 50% of the pairwise comparisons between White Leghorn and each indigenous chicken population (**see Supplementary Table ****S2**). We further selected genomic windows in the top and bottom 2.5% of the distributions of log₂ (θπ_indigenous/θπ_WhiteLeghorn) and (Tajima’s D_indigenous – Tajima’s D_WhiteLeghorn), retaining only those shared by at least 50% of the populations, which resulted in 5,932 (**Supplementary Table ****S3**) and 65,074 (**Supplementary Table ****S4**) significant windows, respectively.

### Runs of homozygosity (ROH)

A total of 1,924 ROH segments were identified across all studied indigenous populations, while commercial population had 4,814 ROH segments (**Supplementary Information**,** Table ****S5**). Due to differing sample sizes between the populations, direct comparison of the absolute number of ROH segments is not appropriate. The identified ROH segments were also classified into three groups based on their length: less than 1 Mb, between 1 and 2 Mb, and more than 3 Mb. Most ROH segments fell into the medium category, the proportion of long ROH segments (> 3 Mb) was individually assessed in all ten populations, with values ranging between 68% and 100%. The total number of ROH segments identified in Iran (Shiraz and Zahedan), Afghanistan, Indonesia (Bali), China (Huaixiang, Huiyang, Huaibeima, Jianghan, Wuhua), and Pakistan chicken populations were 310, 281, 242, 311, 16, 10, 38, 71, 40 and 605, respectively. The number of ROH segments exceeding 3 Mb in length in Afghanistan, Bali, Huaibeima, Jianghan, Pakistan, Shiraz, Wuhua and Zahedan chickens was 43, 38, 2, 4, 70, 19, 1, and 28, respectively. We then identified genes in ROH regions for each breed, allowing genes in ROH common to different breeds to be identified, as well as genes that overlap with significant loci in F_ST_, Tajima’s D, and θπ analyses, and with our previously identified list of heat tolerance–related genes. Genomic inbreeding coefficients based on ROH (FROH) and individual heterozygosity were estimated separately for each population (**Supplementary information**,** Table ****S6**).

### Potential genes, gene set enrichment and pathways analysis in related with heat tolerance

After detecting selective signatures between White Leghorn and the indigenous chicken groups, we annotated the candidate regions. Additionally, annotated protein coding genes consistently identified across these methods for each indigenous population by JVenn^37^ are shown in **Supplementary information**,** Table ****S7****.**

**Fig. 2:**
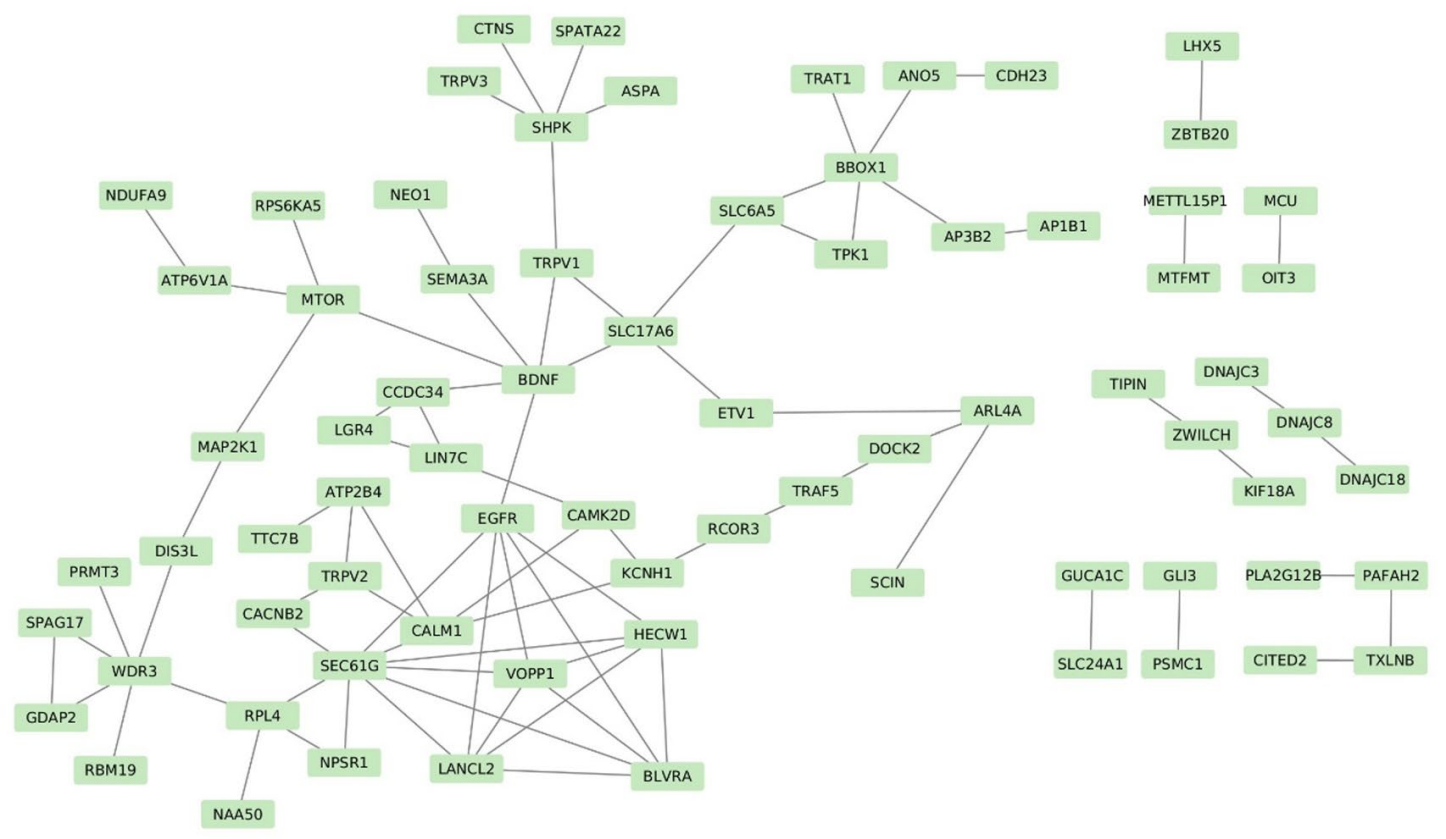
Protein-protein interaction (PPI) network analysis based on identified 113 protein-coding genes that overlap with the selected signature locations and are associated with heat tolerance.

**Fig. 3:**
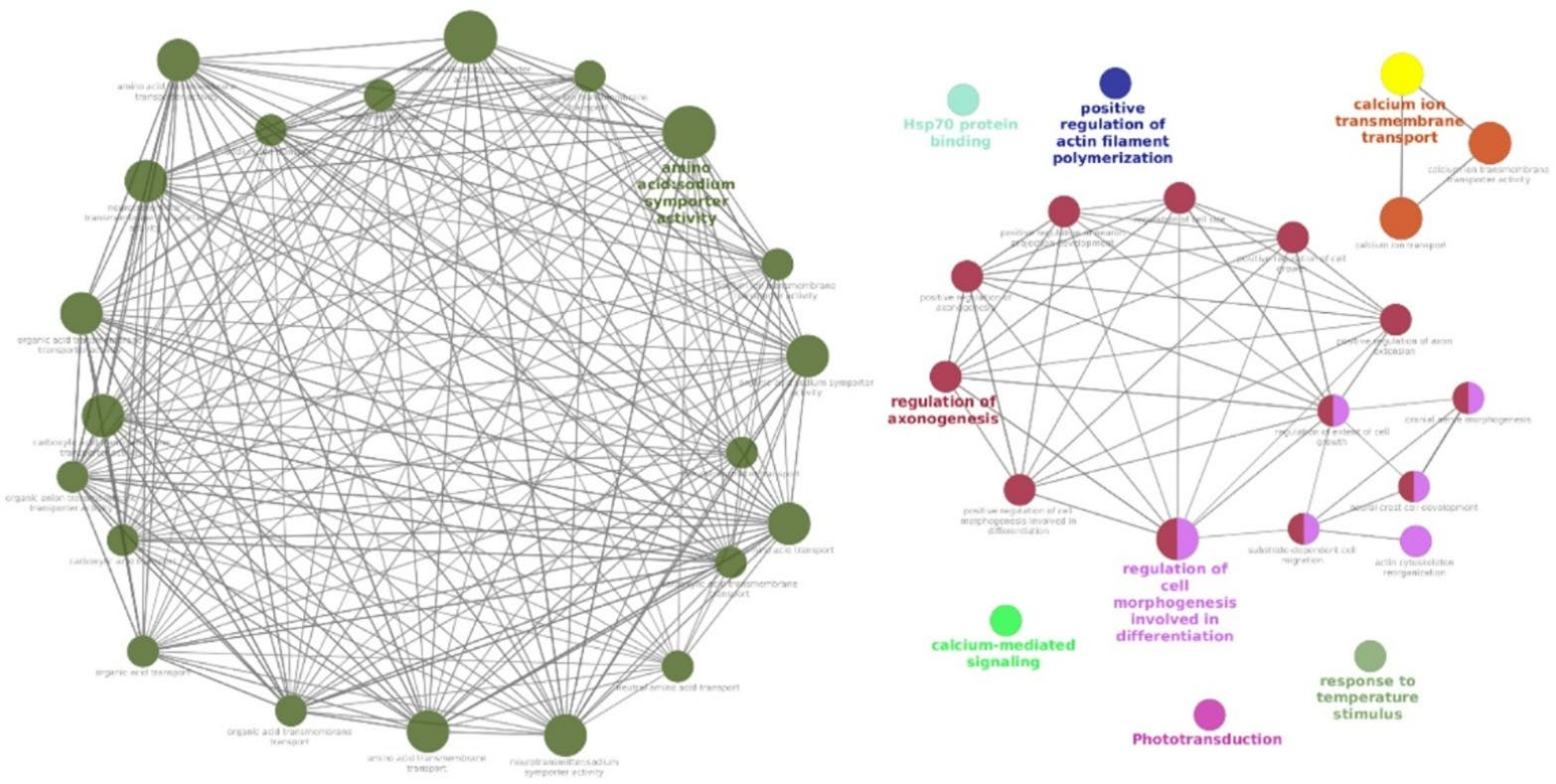
The association between the identified significant terms is enhanced by the 113 protein-coding genes found to overlap with the selected signature locations, all of which are associated to heat tolerance.

Our methods identified numerous candidate genes within high-confidence selected regions potentially linked to heat tolerance, building on our previous findings^31, 32^ and aligning with heat tolerance-associated genes listed in the NCBI database. A total of 267 genes were identified within the convergent selective-sweep windows shared by ≥ 50% of the indigenous populations. Of these, 126 had been previously reported in heat-stress contexts. To focus on the most robust candidates, we applied an additional stringent filter requiring each gene to be supported by at least three of the four statistical methods (F_ST_, θπ ratio, Tajima’s D, ROH) and/or to contain near-fixed alleles (Fisher’s exact test, FDR < 0.05). This reduced the final high-confidence candidate list to 113 genes, The remaining 28 genes, although located in convergent regions, did not fulfil these strict criteria and are listed in **Supplementary Table ****S7**.To investigate the interactions among 113 candidate genes—selected based on ROH and at least two additional methods (F_ST_, Tajima’s D, and θπ)—we used the STRING database for interaction analysis (Fig. 2) and conducted functional enrichment using the ClueGO plugin in Cytoscape (Fig. 3).

Gene set enrichment analysis revealed several significantly enriched terms associated with tolerance to heat stress. Among these, “Hsp70 protein binding” was identified within the molecular function category, while terms such as “calcium-mediated signaling,” “response to temperature stimulus,” “positive regulation of actin filament polymerization,” “calcium ion transport,” “calcium ion transmembrane transport,” and “calcium ion transmembrane transporter activity” were significantly enriched within the biological process category. **(Supplementary information**,** Table ****S8****)**.

### Functional enrichment analysis

We identified 14 genes under selection that are significantly associated with GO terms involved in heat stress, as detailed in **Supplementary Table ****S8****.** These genes include *CDH23*, *NPSR1*, *MCU*, *TRPV2*, *TRPV1*, *TRPV3*, *ATP2B4*, *CALM1*, *CACNB2*, *TRAT1*, *BDNF*, *SCIN*, *WIPF3*, and *PRKD1*.

**Fig. 4:**
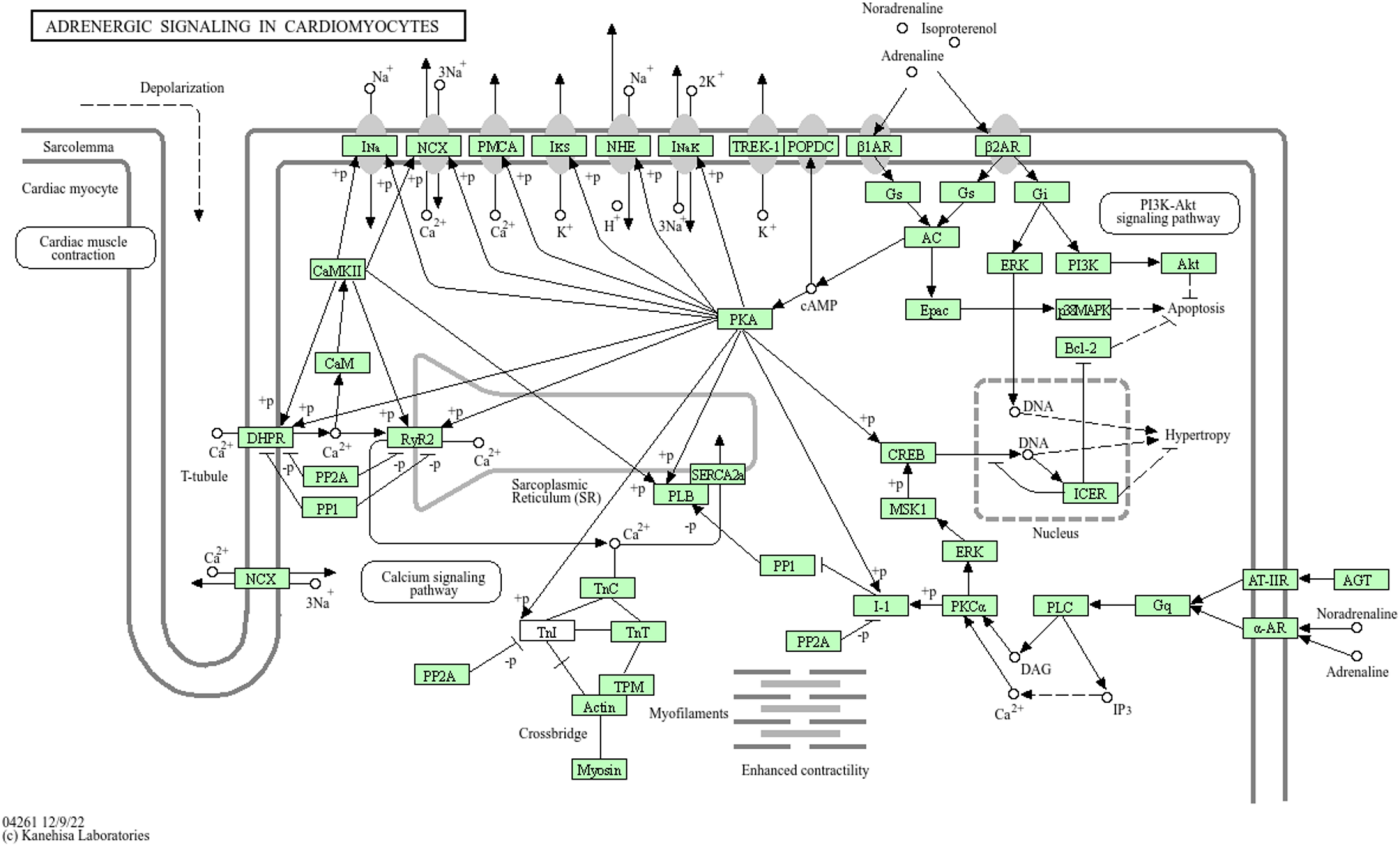
Functional Enrichment Highlights Adrenergic and Calcium Signaling Pathways. Image reproduced with written permission from Kanehisa Laboratories (Ref: 251565)^38, 39, 40^.

The enrichment analysis revealed significant involvement of two KEGG pathways: Adrenergic signaling in cardiomyocytes (*p* = 1.70 × 10⁻²) and the calcium signaling pathway (*p* = 5.10 × 10⁻²). As illustrated in Fig. 3, several of the genes identified in our study are mapped onto the adrenergic signaling pathway. Notably, *CALM1* (calmodulin, CaM) and *ATP2B4* (PMCA) are directly involved in calcium regulation and signal transduction within cardiomyocytes (Fig. 4). *CALM1* activates downstream targets such as *CaMKII*, while *ATP2B4* facilitates calcium efflux. Although not shown in the pathway diagram, *TRPV1*, *TRPV2*, and *TRPV3* are thermosensitive calcium channels known to participate in the calcium signaling pathway and contribute to heat stress response. *PRKD1* is also connected to *PKC* signaling downstream of adrenergic activation. Additional genes such as *BDNF* and *CACNB2*, while not visualized in this diagram, are associated with neurotrophic and calcium channel functions. Genes including *MCU*, *CDH23*, *NPSR1*, *TRAT1*, *SCIN*, and *WIPF3* are not represented in the current KEGG pathway map.

**Fig. 5:**
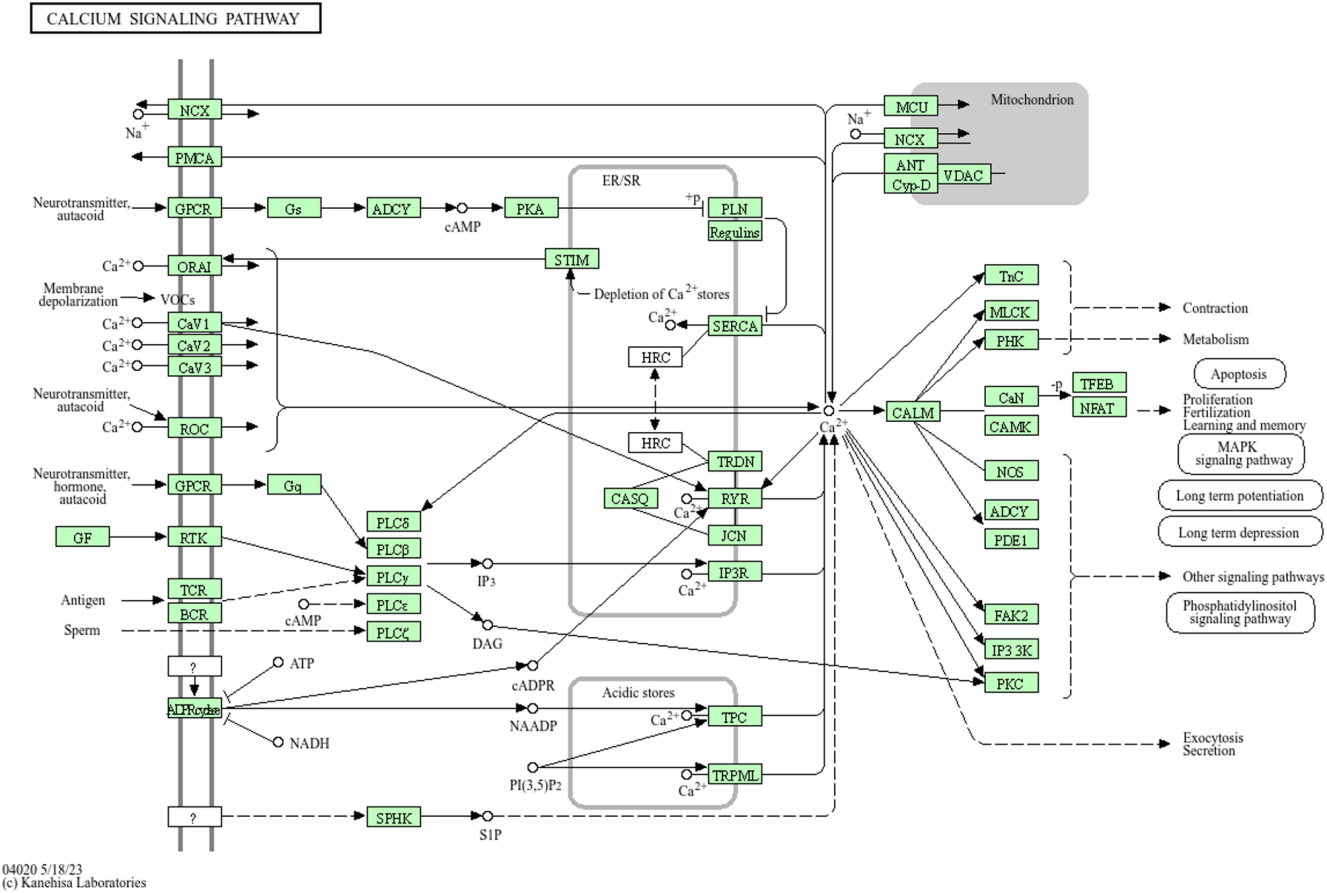
Key genes involved in the calcium signaling pathway. Highlighted genes are associated with various downstream cellular functions. Image reproduced with written permission from Kanehisa Laboratories (Ref: 251565)^38, 39, 40^.

Analysis of the calcium signaling pathway (Fig. 5) revealed the involvement of several key genes, including *CDH23*, *NPSR1*, *MCU*, *TRPV1*, *TRPV2*, *TRPV3*, *ATP2B4*, *CALM1*, *CACNB2*, *TRAT1*, *BDNF*, *SCIN*, *WIPF3*, and *PRKD1*, which contribute to distinct downstream cellular processes. *MCU* and *ATP2B4* regulate mitochondrial and cytoplasmic calcium homeostasis, influencing apoptosis and energy metabolism. *CALM1*, a central calcium sensor, mediates calcium-dependent activation of signaling molecules such as CaMK and CaN, linking calcium influx to memory, learning, and synaptic plasticity. Ion channels *TRPV1–3* and *CACNB2* modulate calcium entry in response to depolarization or sensory stimuli, which are critical for neuronal excitability and neurotransmitter release. *BDNF* expression, regulated by calcium signaling, was implicated in synaptic strengthening and neuroplasticity. *PRKD1* acts downstream to regulate secretion and cell survival pathways. Immune-related gene *TRAT1* and receptor *NPSR1* mediate calcium-dependent activation in immune and stress responses. Structural regulators *SCIN* and *WIPF3* may facilitate actin remodeling and exocytosis in response to calcium flux. Collectively, the activation of these genes, as shown in Fig. 5, underscores the central role of calcium signaling in orchestrating diverse biological processes such as neurotransmission, immune activation, cellular secretion, and gene expression, all of which contribute to the cellular response and adaptation to heat stress^41, 42^–^43^.

### The fixation of alleles in candidate genes associated with heat tolerance

After detection of 14 selected genes associated with heat tolerance, we focused on putatively functional variants in these genes. To assess the genotype distribution and fixation patterns between the indigenous and White Leghorn populations, we analyzed the frequencies of homozygous reference (0/0), heterozygous (0/1), and homozygous alternate (1/1) genotypes. The differential analysis revealed decreases or increases in the percentage of 1/1 or 0/0 genotypes in the White Leghorns compared to the native population, or vice versa. These findings highlight substantial changes that likely reflect underlying genetic and selective pressures acting on the two populations. We discovered 732 SNPs that showed near-complete fixation across all comparisons. Among these, 721 were uniquely fixed in the White Leghorn population, while only 11 were nearly fixed in the indigenous populations. The majority of these indigenous-specific SNPs were situated in intronic regions, though a few exhibited coding variants. The allele frequency distribution for each population is provided in **Supplementary Information**,** Figure ****S2**. Notably, the SNP rs3386031003 at position 5:3943152 is a missense mutation, while rs3386050400 at position 5:3952604 is an upstream gene variant; both are situated within the *BDNF* gene. Additional details are provided in **Supplementary Information**,** Table ****S9**. We identified a set of SNPs that were fixed in over 70% of pairwise comparisons between White Leghorn and several indigenous populations. Notably, the SNP rs1060165215 located at position 5:33441126, a missense variant in the *PRKD1* gene, was fixed in the following populations, along with their respective FDR-adjusted p-values: Afghanistan (*p* = 3.0163E-05), Bali (*p* = 9.4671E-07), Huaibei (*p* = 7.43893E-05), Huaixiang (*p* = 7.92188E-05), Huiyang (*p* = 8.11157E-05), Jianghan (*p* = 8.5849E-05), Shiraz (*p* = 5.17514E-07), Wuhua (*p* = 6.32765E-09), and Zahedan (*p* = 5.36687E-08). Additionally, the SNP rs3387803766 at position 5:33330346, located in the splice region in intron 16 of *PRKD1*, was fixed in Afghanistan (*p* = 2.46038E-05), Bali (*p* = 7.82256E-07), Huaibei (*p* = 7.30574E-05), Huaixiang (*p* = 7.77693E-05), Huiyang (*p* = 7.99212E-05), Jianghan (*p* = 8.45744E-05), Wuhua (*p* = 2.96E-09), and Pakistan (*p* = 7.88122E-12). Detailed results are provided in **Supplementary Information**,** Table ****S10**.

## Discussion

One of the major strengths of our study was the use of independent pairwise comparisons between the White Leghorn breed and each of the ten genetically, geographically and climatically diverse indigenous populations (arid-hot regions of Iran, Afghanistan, and Pakistan; humid-tropical Bali; subtropical southern China). Rather than pooling all indigenous birds, we deliberately required candidate selective sweep regions and genes to appear in at least 50% of these pairwise comparisons (i.e., shared across a minimum of five to six populations representing contrasting climatic zones). This stringent, convergence-focused threshold was chosen to retain only the most robust and repeatedly selected signals of heat-stress adaptation, thereby minimizing false positives arising from population-specific drift or local demographic events.

This approach ensured that the reported heat-tolerance candidate genes (e.g., *TRPV1*, *TRPV2*, *TRPV3*, *ATP2B4*, *MCU*, *CACNB2*, *CALM1*) and enriched pathways reflect true convergent evolution across a broad thermal gradient (THI ranging from 72.1 in Zahedan to 83.4 in Pakistan). These patterns indicate that, while a core molecular toolkit is remarkably conserved across indigenous chickens, additional locally optimized mechanisms have evolved to complement the shared adaptive response under the most severe and heat stress.

In this study, we identified candidate heat tolerance associated genes under selection. These include *CDH23*, *NPSR1*, *MCU*, *TRPV2*, *TRPV1*, *TRPV3*, *ATP2B4*, *CALM1*, *CACNB2*, *TRAT1*, *BDNF*, *SCIN*, *WIPF3*, and *PRKD1*. Selected genes were identified based on ROH and at least two of three population genetic metrics: F_ST_, Tajima’s D, and θπ. This combined approach provides strong evidence of adaptive evolution in response to environmental heat stress^44^^45^,.

GO enrichment analysis showed that these candidate genes cluster significantly in three biological themes: calcium signaling, thermosensation, and neurodevelopmental plasticity. These processes are key for cellular and behavioral adaptation to heat stress. Calcium-related processes, including calcium ion transport (GO:0006816) and calcium-mediated signaling (GO:0019722), were enriched. Also *ATP2B4*, *MCU*, *CALM1*, and *TRPV1–3*, play central roles in these pathways. This highlights an evolutionarily conserved mechanism that maintains calcium balance under heat stress. Experimental studies support these findings. For example, *ATP2B4* (*PMCA4*) is crucial for calcium homeostasis during hyperthermia^46^^47^,, while *MCU* regulates mitochondrial calcium uptake and heat-induced apoptosis^48^.

The *TRPV* ion channels, especially *TRPV1*, *TRPV2*, and *TRPV3*, are key thermosensors under selection. *TRPV1* activates at approximately 43°C. It enhances peripheral heat sensation and mediates central cooling in the medial preoptic area)mPOA(. Its role has been demonstrated in desert rodents, which show altered thermosensitivity^49^. In mice, *TRPV1* activation boosts antioxidant defenses and reduces inflammation under heat stress^50^. *TRPV2* responds to temperatures above 52 °C, detecting extreme heat^51^, while *TRPV3* is active at 33–39 °C in keratinocytes. It links thermal signals to calcium pathways, induces HSP expression via calmodulin/CaMK, and supports skin repair^52, 53, 54, 55^–^56^.

*CALM1* plays a key role in calcium signaling activating kinases such as CaMKs and phosphatases like calcineurin following heat-induced calcium influx. These enzymes regulate heat shock protein expression, inhibit apoptosis, and stabilize intracellular proteins^57^^58^,. Together, these actions enhance cellular resilience to heat stress.

Genes such as *BDNF*, *CACNB2*, *PRKD1*, and *TRAT1* also contribute to the broader stress adaptation framework. Although *BDNF* is not directly annotated in KEGG pathways, it plays a well-established role in calcium-dependent synaptic plasticity and neuroprotection, acting through complex signaling networks beyond those currently captured by KEGG databases^59^^60^,. *PRKD1* acts downstream of PKCµ to regulate α-catenin phosphorylation and maintain endothelial barrier integrity in response to IL-33 under hypoxic conditions^61^. *TRAT1* and *NPSR1* participate in immune-related calcium signaling, which may connect heat stress to inflammatory and immune pathways^41^^42^,. *SCIN* and *WIPF3*, as cytoskeletal regulators, might facilitate calcium-dependent actin remodeling and vesicle trafficking, thereby enhancing cellular adaptability^62^^63^,.

Taken together, these findings show that calcium signaling is a central integrative axis in the heat stress response^43^ – it links thermosensory input from *TRPV* channels, calcium homeostasis mediated by *ATP2B4*, *MCU*, and *CACNB2*, and downstream signaling cascades involving *CALM1* and *PRKD1*. These pathways promote protective cellular functions such as protein stabilization, apoptosis inhibition, and immune regulation^64, 65, 66^–^67^. The overlap of positive selection signals and functional enrichment within this network provides strong evidence for evolutionary adaptation aimed at maintaining physiological homeostasis under high-temperature conditions^68^.

In White Leghorn chickens, many alleles related to thermosensation, calcium signaling, and cellular stress responses were fixed or nearly fixed (AF ≅ 1). In contrast, these variants were rare or absent in indigenous populations (AF = 0 to 0.08). For instance, we identified fixed variants located in intronic and downstream regions of the *TRAT1* gene. Similarly, intronic and upstream variants in *CACNB2*—a gene involved in calcium channel activity and neural development—were fixed in both Leghorn and indigenous chicken breeds. In Leghorns, fixed variants were also found in the intron, downstream region, and 3’ UTR of *WIPF3*, which is associated with actin cytoskeleton organization and stress response, as well as intronic variants in *NPSR1*, a gene linked to neuropeptide signaling and immune system regulation. Fixed variants in *CDH23*, a gene essential for mechanosensation, were detected across several genomic regions, including intronic, splice sites, synonymous positions, upstream, downstream, and the 3’ UTR. We also observed variants in the intronic, downstream, and 3’ UTR regions of *PRKD1*, which may influence the heat shock response. Moreover, a fixed variant in *MCU*—present in the 3’ UTR, 5’ UTR, upstream area, intron, and including a missense mutation—was found in Leghorns; *MCU* plays a critical role in regulating mitochondrial calcium uptake. The fixation of these alleles in Leghorns suggests that artificial selection targeting productivity traits might have reduced thermotolerance. Conversely, fixed intronic variants were identified in *CACNB2*, *TRPV3*, and *PRKD1*, alongside missense and upstream variants in *BDNF* in indigenous chickens. These native breeds appear to have retained more ancestral alleles, likely due to natural adaptation to hot and humid climates. Collectively, these genes stand out as promising candidates for functional studies and selective breeding to improve heat tolerance in commercial poultry.

Intronic variants, particularly those located near splice sites, have recently gained significant attention as key regulators of gene expression and post-transcriptional modifications. These variants can induce events such as intronic polyadenylation (IPA), intron retention, or splice site alterations, leading to the production of alternative RNA isoforms and ultimately altering gene expression—without necessarily affecting coding regions. While the functional roles of intronic variants, including intronic polyadenylation (IPA), have been well-documented in human cancer and economically important traits in cattle^69, 70, 71^–^72^, no studies to date have explored their role in the heat stress response in chickens despite the fact that heat stress represents one of the most critical economic and biological challenges in the global poultry industry. In this study, we identify for the first time intronic variants including ones in heat-responsive genes such as *WIPF3*, *TRAT1*, and *CACNB2*, which may influence gene expression through mechanisms such as intronic polyadenylation (IPA) or splice site alterations. Given the strong conservation of RNA processing mechanisms across vertebrates, our findings provide a foundation for future functional investigations (e.g., minigene assays) to assess the direct role of these variants in modulating heat stress responses in indigenous chicken breeds.

This study has several limitations. Firstly, we used THI as a population-level proxy for heat stress that may not capture individual heat exposure of microclimatic variation. Secondly, inference of selection from population-genetic statistics can be confounded by demographic history and population structure – something which may be particularly apparent in a domesticated species like the chicken. By way of mitigation, we used a strategy requiring identification of relevant genes using several independent methods for detecting selection to minimize false positives. Thirdly, we relied on short read data which is unlikely to have resolved any structural variations, limiting us to identifying SNPs and short INDEL variants. Finally, although our work identifies strong candidates for heat stress adaptation, further work is required to functionally validate our findings and establish a causal link between polymorphisms and adaptation to heat.

## Conclusion

Our findings underscore the potential functional relevance of regulatory variants—particularly intronic ones—in shaping the transcriptional response to heat stress in chickens. This study is the first to highlight the possible involvement of non-coding variants in thermal adaptation in poultry. Candidate genes such as *PRKD1*, *CDH23*, *WIPF3*, *TRAT1*, and *CACNB2* harbor variants that may act through mechanisms like intronic polyadenylation or splicing disruption. These discoveries lay the groundwork for future experimental validation and breeding strategies aimed at enhancing heat tolerance in indigenous breeds. Further functional assays will be crucial to unravel the exact molecular pathways these variants modulate under thermal stress.

## Supplementary Information

Below is the link to the electronic supplementary material.

Supplementary Material 1

Supplementary Material 2

Supplementary Material 3

Supplementary Material 4

Supplementary Material 5

Supplementary Material 6

Supplementary Material 7

Supplementary Material 8

Supplementary Material 9

Supplementary Material 10

Supplementary Material 11

Supplementary Material 12

## Data Availability

All data used in this study are publicly available from online repositories. Table S1 contains accession numbers for all samples.
