# Supplementary figures and images for: Whole genome analysis of selection associated with resistance to heat stress in chickens
Source: Sci Rep. 2026 Apr 7;16:11726. doi: 10.1038/s41598-026-41813-8 (PMC13062120; doi:10.1038/s41598-026-41813-8)

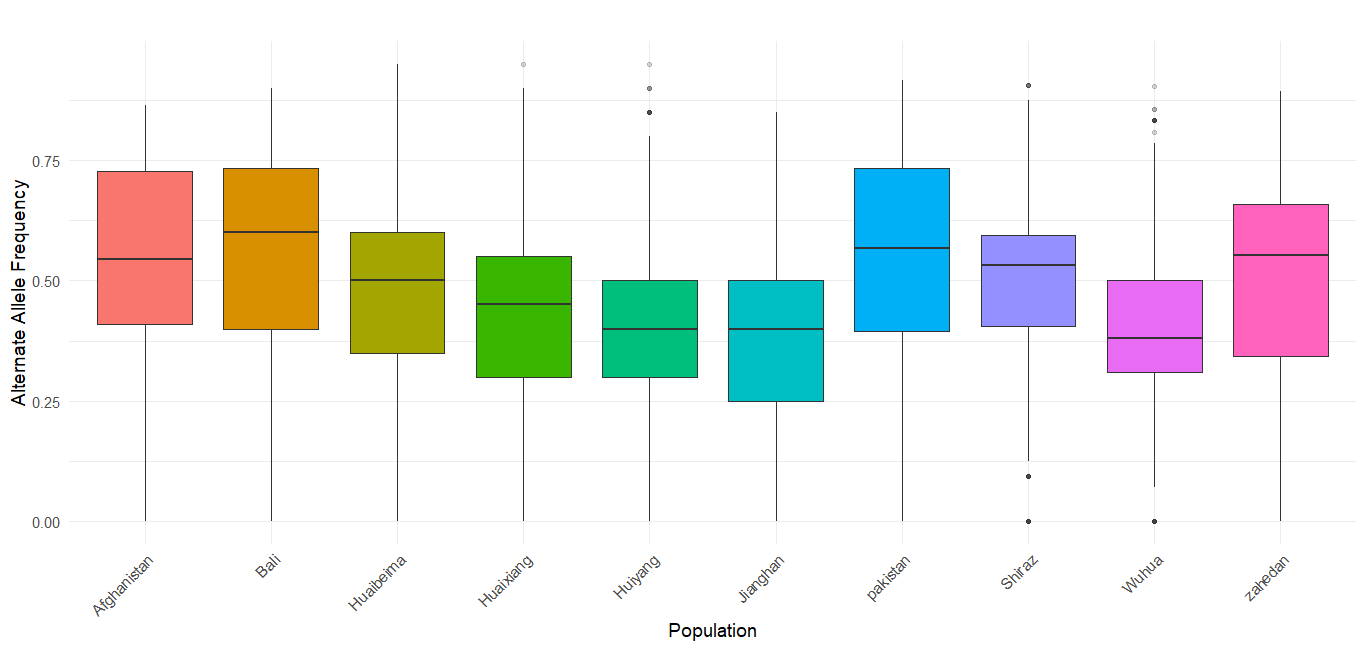

Supplement: Supplementary file 1 — Supplementary Material 1 [file 41598_2026_41813_MOESM1_ESM.png]
