# Supplementary material for: Whole genome analysis of selection associated with resistance to heat stress in chickens
Source: Sci Rep. 2026 Apr 7;16:11726. doi: 10.1038/s41598-026-41813-8 (PMC13062120; doi:10.1038/s41598-026-41813-8)
Supplement: Supplementary file 4 — Supplementary Material 4 [file 41598_2026_41813_MOESM4_ESM.docx]

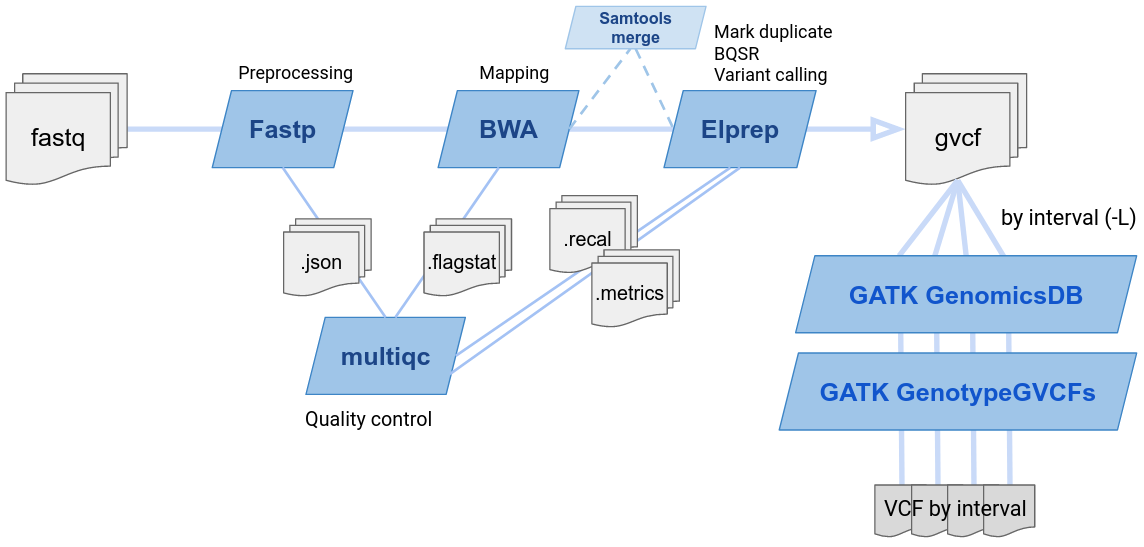


**Supplementary information, Fig. S1.** Processing Pipeline: To maintain consistency across samples, all datasets were re-processed starting from the raw fastq reads. The workflow included pre-processing and quality control of the reads, followed by alignment to the chicken genome assembly GRCg6b, variant calling, and the generation of a VCF file.


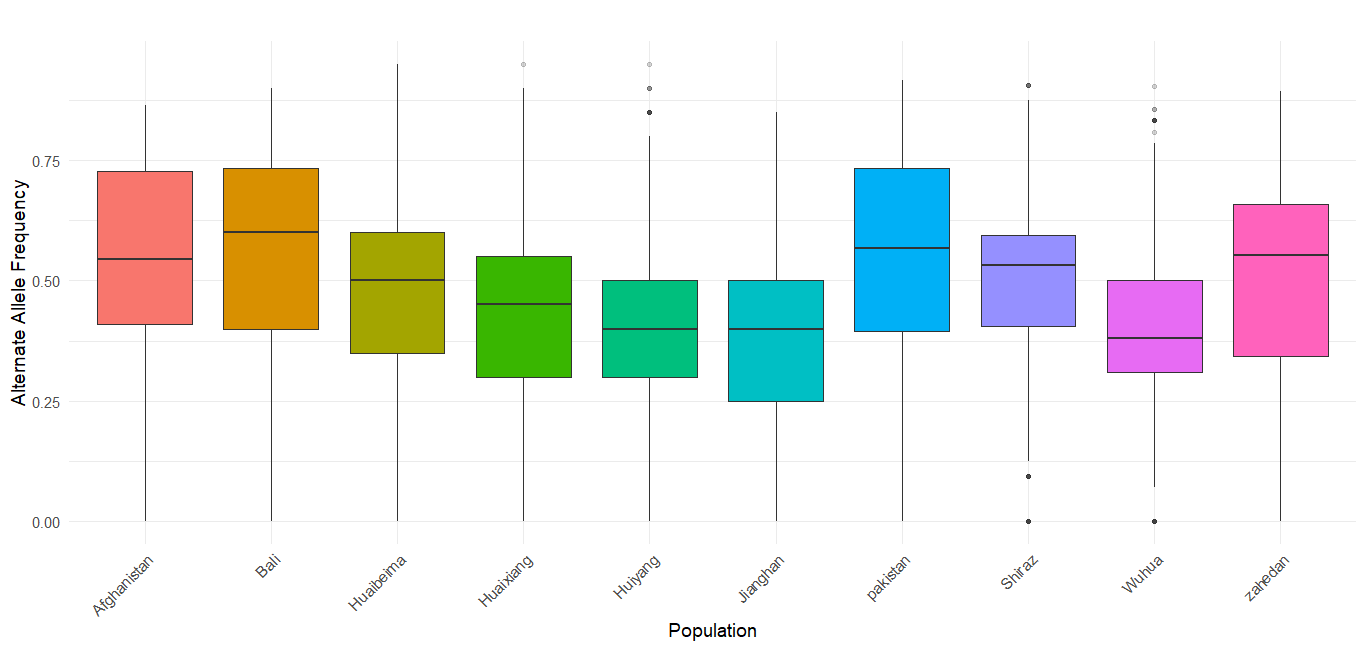


**Supplementary information, Fig. S2.** Distribution of Alternate Allele Frequency per Population.
